# Collagen-Based Tissue Engineering Strategies for Vascular Medicine

**DOI:** 10.3389/fbioe.2019.00166

**Published:** 2019-07-12

**Authors:** Francesco Copes, Nele Pien, Sandra Van Vlierberghe, Francesca Boccafoschi, Diego Mantovani

**Affiliations:** ^1^Laboratory for Biomaterials and Bioengineering, Canada Research Chair Tier I for the Innovation in Surgery, Department of Min-Met-Materials Engineering & Regenerative Medicine, CHU de Quebec Research Center, Laval University, Quebec City, QC, Canada; ^2^Laboratory of Human Anatomy, Department of Health Sciences, University of Piemonte Orientale, Novara, Italy; ^3^Polymer Chemistry & Biomaterials Group, Department of Organic and Macromolecular Chemistry, Centre of Macromolecular Chemistry, Ghent University, Ghent, Belgium

**Keywords:** collagen, tissue engineering, cardiovascular, coating, drug delivery system, vascular model

## Abstract

Cardiovascular diseases (CVDs) account for the 31% of total death per year, making them the first cause of death in the world. Atherosclerosis is at the root of the most life-threatening CVDs. Vascular bypass/replacement surgery is the primary therapy for patients with atherosclerosis. The use of polymeric grafts for this application is still burdened by high-rate failure, mostly caused by thrombosis and neointima hyperplasia at the implantation site. As a solution for these problems, the fast re-establishment of a functional endothelial cell (EC) layer has been proposed, representing a strategy of crucial importance to reduce these adverse outcomes. Implant modifications using molecules and growth factors with the aim of speeding up the re-endothelialization process has been proposed over the last years. Collagen, by virtue of several favorable properties, has been widely studied for its application in vascular graft enrichment, mainly as a coating for vascular graft luminal surface and as a drug delivery system for the release of pro-endothelialization factors. Collagen coatings provide receptor–ligand binding sites for ECs on the graft surface and, at the same time, act as biological sealants, effectively reducing graft porosity. The development of collagen-based drug delivery systems, in which small-molecule and protein-based drugs are immobilized within a collagen scaffold in order to control their release for biomedical applications, has been widely explored. These systems help in protecting the biological activity of the loaded molecules while slowing their diffusion from collagen scaffolds, providing optimal effects on the targeted vascular cells. Moreover, collagen-based vascular tissue engineering substitutes, despite not showing yet optimal mechanical properties for their use in the therapy, have shown a high potential as physiologically relevant models for the study of cardiovascular therapeutic drugs and diseases. In this review, the current state of the art about the use of collagen-based strategies, mainly as a coating material for the functionalization of vascular graft luminal surface, as a drug delivery system for the release of pro-endothelialization factors, and as physiologically relevant *in vitro* vascular models, and the future trend in this field of research will be presented and discussed.

## Introduction and Short Historical Perspective

Cardiovascular diseases (CVDs) account for 17.9 million deaths each year, making them the leading cause of death in the world (WHO^1^). Heart attacks and strokes account for 85% of these deaths. Most often, atherosclerosis is at the basis of these two pathologies. Atherosclerosis is a pathological progressive condition in which plaques, mainly due to the accumulation of lipids, cholesterol, foamy cells, and cellular debris, progressively grow inside the lumens, thus leading to the partial or complete obstruction of blood flow, and leading to severe medical conditions and, ultimately, to death. The increase of risk factors associated with the pathology (obesity, diabetes, hypertension, and smoking), coupled with the increase in average life expectancy, has led to the urgent search for durable and effective solutions. Vascular bypass/substitution surgery represents the most common, ultimate clinical treatment of occlusive CVDs. Autologous blood vessels, such as saphenous veins or radial arteries, that present the best structural, mechanical, and biological properties are the gold standard for this kind of application. However, the use of these substitutes is not always possible, due to the multiple surgical procedures required, or the poor general health conditions of patients. Some of the limiting factors for the use of autografts include the typical old age of the patients needing treatments, vascular diseases preventing the use of autologous vessels, and/or previous harvesting for other surgical treatments. In this light, the need for other sources of vascular substitutes is critically urgent. Synthetic prostheses development started in the 1950s and opened a therapeutic alternative for the replacement of injured arterial segments. The first synthetic vascular bypass has been performed in 1952 with the implantation of a porous textile prosthesis made of polyethylene terephthalate (PET), also known as Dacron® (Voorhees et al., 1952; Kannan et al., 2005). Prostheses made of Dacron® are usually applied for the replacement of vessels of large caliber (>10 mm in diameter). Then, in 1976, the first use of expanded polytetrafluoroethylene (ePTFE), also known as Teflon®, was reported (Kannan et al., 2005; Chlupac et al., 2009). These prostheses are applied in the replacement of medium-sized vessels, between 6 and 10 mm in diameter. No studies show the superiority of PET compared to ePTFE (Roll et al., 2008). Since their introduction in cardiovascular medicine, a number of improvements have been made to enhance the performance of the synthetic vascular substitutes (SVS). Nevertheless, their low patency owing to short- and intermediate-term failure still limits their clinical application. Two of the main causes of SVS failure are thrombosis and intima hyperplasia. In-graft thrombosis is the result of a perturbation of the hemostatic balance, usually maintained by a series of anti-coagulation reactions involving both physical–mechanical and biological factors, acting on the inhibition of the coagulation process (Edelberg et al., 2001). Among the different factors acting in this complex balance, the intima layer, composed of endothelial cells (ECs), greatly contributes to the maintenance of the hemostatic balance by producing several antithrombotic molecules. The disruption of the endothelial layer or its absence greatly compromises the antithrombotic environment of healthy blood vessels. Intimal hyperplasia, especially at the anastomotic sites, results in the abnormal migration and proliferation of vascular smooth muscle cells (SMCs) with associated deposition of extracellular connective tissue matrix and is thought to be due to a variety of injuries that always involve some endothelial damage (Clowes, 1993). Intima hyperplasia is composed of about 20% of vascular SMCs that have migrated from the media to the intima and have proliferated and deposited extracellular matrix (ECM), which comprises most (60–80%) of the intimal area. Normal endothelium produces factors that inhibit SMC proliferation. A damage of the endothelium layer decreases the production of growth-inhibiting factors and increases the expression of growth-stimulating factors, shifting the balance toward SMC proliferation and migration toward the intima.

As previously described, both these adverse outcomes have a common basis in the lack or uncomplete endothelialization of the implanted substitutes. Therefore, the rapid establishment of a complete and functional ECs layer on the luminal surface of SVS would be beneficial to prevent failures and for ensuring the long-term patency of the implanted substitutes.

Tissue engineering is a multidisciplinary domain aimed to develop biologically based tissues that can be used in the clinical treatments of diseases. Tissue engineering products have already shown to be effective in different applications, ranging from burn treatment to osteo-regeneration. The success obtained by this approach in other medical fields has opened the door for its use in vascular reconstruction. The use of scaffolding systems based on natural polymers is one of the strategies used in vascular tissue engineering (vTE) to promote cellular integration and proliferation. The ideal scaffold should be able to mimic the native vascular ECM and the highly complex organization of the arterial wall, showing important biological and mechanical characteristics, such as non-thrombogenicity, hemocompatibility, biocompatibility (low cytotoxicity, optimal cell adhesion, bioresorbable), and non-immunogenicity, along with tensile strength and viscoelasticity.

Among the natural polymers currently used for vTE, collagen is the most used one. Collagen is one of the main components of the vascular ECM. Its main function is to subdue constraints imposed by elongation under pressure in large vessels while providing attachment for vascular cells [12].

**Figure 1:**
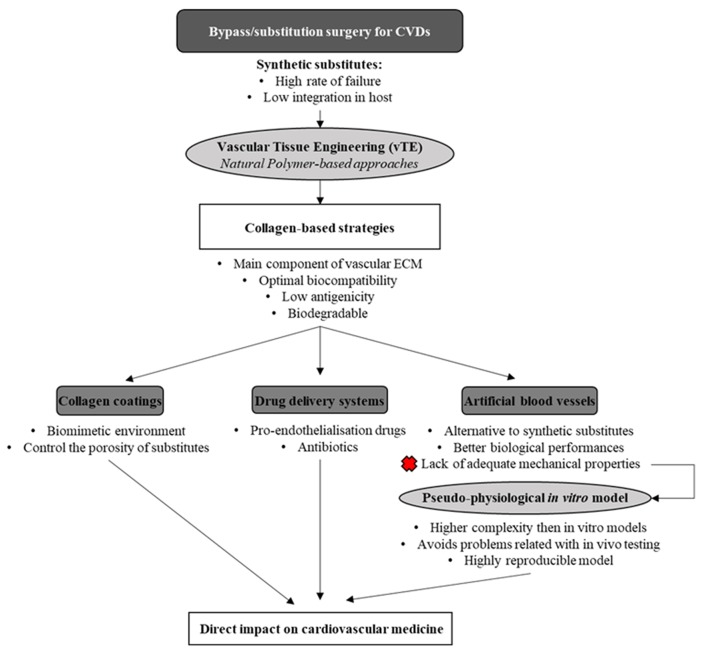
Schematic view of the layout of this review.

In this review, the main properties of the collagen molecule, along with the different types, will be presented. Moreover, collagen-based coatings will be detailed mainly in the context of vascular substitutes, and the use of collagen for the development of drug delivery systems (DDS) (with a focus on the ones with vascular applications) will be discussed. Finally, the development of *in vitro* physiologically relevant artery models based on collagen scaffolds for the study and validation of drugs and cardiovascular devices will be overviewed (Figure 1).

## Collagen

### Structure and Biosynthesis

Collagen is the most abundant protein in animals, including the human body (Shoulders and Raines, 2009). It accounts for one third of the total protein content, and it constitutes the main component of the ECM. To date, 28 different collagen types have been identified in vertebrates, and the discovery of collagen in dinosaur bone fossils make it the oldest protein ever detected (Exposito et al., 2002; Schweitzer et al., 2007). Collagens can be divided into two main categories: fibrillar and non-fibrillar. Fibrillar collagens form elongated fibril structures, which are known for their structural role in mechanical support for most animal tissues (Hulmes, 2002; Jenkins et al., 2005; Exposito et al., 2010). Non-fibrillar collagens can be divided in sub-categories, such as network-forming collagens (collagen types IV and VII), fibril-associated collagens with interrupted triple helices (FACITs, collagen types IX and XII), and membrane-associated collagens with interrupted triple helices (MACITs). The main types of collagens, along with their distribution and composition, are listed in Table 1.

**Table 1:** Main collagen types and their distribution in the human body.

**Structure**	**Type**	**Composition**	**Chains**	**Distribution**
Fibrillar Collagens	I	Heterotrimer	[α1(I)]_2_α2(I)	Skin, cornea, blood vessels, bone, ligaments, and tendons
	II	Homotrimer	[α1(II)]_3_	Cartilage, intervertebral discs
	III	Homotrimer	[α1(III)]_3_	Skin, blood vessels
	V	Heterotrimer	[α1(V)]_2_α2(V) or α1(V)α2(V)α3(V)	Skin, cornea, blood vessels, bone, ligaments, and tendons
	XI	Heterotrimer	α1(XI)α2(XI)α3(XI)	Cartilage, intervertebral discs
FACITs	IX	Heterotrimer	α1(IX)α2(IX)α3(IX)	Cartilage
	XII	Homotrimer	[α1(XII)]_3_	Ligaments and tendons
Network Forming	IV	Heterotrimer	[α1(IV)]_2_α2(IV)	Basal lamina
	VI	Heterotrimer	α1(VI)α2(VI)α3(VI) or α1(VI)α2(VI)α4(VI)	Bone, cartilage, cornea, dermis
	VII	Homotrimer	[α1(VII)]_3_	Under stratified epithelium
MACITs	XIII	—	—	Endothelial cells, dermis, eye, heart

*Modified from Shoulders and Raines (2009). FACITs, fibril-associated collagens with interrupted triple helices; MACITs, membrane-associated collagens with interrupted triple helices*.

**Figure 2:**
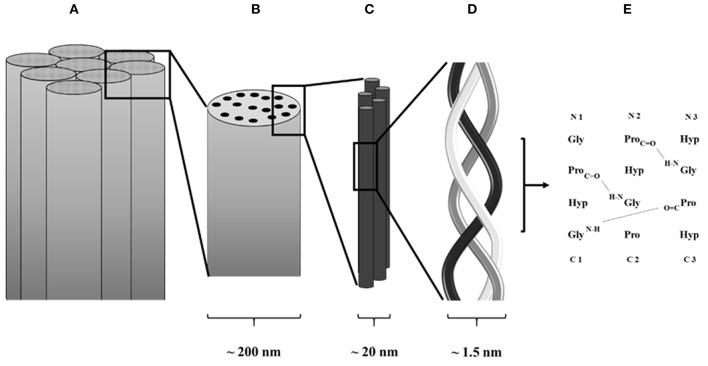
Schematic collagen structure. **(A)** Collagen fiber formed by assembled collagen fibrils. **(B)** Collagen fibrils. **(C)** Assembled tropocollagen. **(D)** Collagen triple helix. **(E)** Hydrogen bond in between collagen α chains.

All collagens, fibrillar or not, are characterized by the same molecular structure, which is composed of three α chains. These chains can either be identical, thus originating a homotrimer, or be a combination of two or three distinct α chains forming a heterotrimer. Each α chain contains three basic amino acids, which are glycine, proline, and hydroxyproline, and is characterized by the presence of at least one collagenous domain, consisting of a repeating Gly-Xaa-Yaa triplet (Brazel et al., 1987), where Xaa is usually a proline and Yaa is a hydroxyproline. However, both Xaa and Yaa can be any amino acid, conferring specific functions for the collagen (Figure 2).

Fibrillar collagens are the most used in the production of collagen-based biomaterials, with type I being the most abundant collagen type in the human body (Di Lullo et al., 2002). During the synthesis of fibrillar collagen molecules, alpha chains are formed by ribosomes present on the surface of rough endoplasmic reticulum (RER). These chains present registration peptides and a signal peptide that, once released in the lumen of the RER, is cleaved to form pro-collagen chains (Ishikawa and Bachinger, 2013). At this point, the pro-collagens go through several modifications (mainly hydroxylation of the lysine and proline residues and glycosylation of specific hydroxylysines) and they are finally assembled in triple helical structures. These pro-collagen triple helices are then transferred to the Golgi apparatus to be encapsulated and secreted by exocytosis. Once in the extracellular environment, the registration peptides present on the pro-collagen are cleaved and tropo-collagen is formed. Through cross-linking, several tropo-collagen molecules are assembled to produce collagen fibrils. In turn, collagen fibrils assemble to form collagen fibers (Bella and Hulmes, 2017).

### Collagen as a Biomaterial

Collagen is the most used natural polymer for tissue engineering applications due to its presence in the ECM of almost every human tissue. The use of collagen as a biomaterial dates back to the early decades of the twentieth century, when the first characterization of the interaction between cells and extracted collagen was studied (Huzella and Lengyel, 1932; Ehrmann and Gey, 1956). The use of collagen is prompted by several characteristics that make it a good material for biomedical applications: Weak antigenicity and robust biocompatibility (Schmitt et al., 1964; Furthmayr and Timpl, 1976; Lee et al., 2001; Lynn et al., 2004), promotion of cell adhesion through cell receptors that recognize a specific peptide sequence within collagen molecules (Gullberg et al., 1992; Smethurst et al., 2007; Konitsiotis et al., 2008), and biodegradability (Chiang et al., 1978; Postlethwaite et al., 1978; Yannas et al., 1982). As an added value, collagen can be isolated from several sources, being one of the most abundant and best conserved proteins among vertebrates. Usual sources for collagen extractions are bovine skin and tendons (Rodrigues et al., 2003), porcine acellular bladder collagen (Chen et al., 1999), porcine collagen type I (Salamanca et al., 2018), and rat tail tendons (Ehrmann and Gey, 1956; Chandrakasan et al., 1976; Habermehl et al., 2005), but collagen has also been extracted from other organisms, such as sponges (Exposito et al., 1991), fishes (Sugiura et al., 2009), kangaroos (Johnson et al., 1999), and alligators (Wood et al., 2008), making it a cost-effective solution for scaffold-based tissue engineering.

Collagen-based biomaterials are mainly used for the treatment of burns and as wound dressing (Chattopadhyay and Raines, 2014). Due to their structure, porosity and surface properties, collagen sponges have long been used for wound dressing applications (Abramo and Viola, 1992; Fleck and Simman, 2010). Moreover, they can be loaded with therapeutic agents, such as growth factors (Lee, 2005) or antibiotics (Sripriya et al., 2004) that greatly improve the healing process once implanted. Another common application for collagen products is as an osteogenic scaffold and filling material in orthopedy (Matassi et al., 2011; Zhang et al., 2018). Collagen type I scaffolds modified with hydroxyapatite have been used as an osteochondral scaffold to improve bone and cartilage regeneration (Kon et al., 2011). Collagen scaffolds can also be used as injectable mineralized bone substitutes (Stephan et al., 2000). Next to this, collagen has been widely used for dentistry applications, such as for the production of membranes for periodontal and implant therapy to improve cell proliferation (Patino et al., 2002). Another field of application for collagen is in ophthalmology as corneal shield (Willoughby et al., 2002; Eshar et al., 2011) and as eye implants for post-operative recovery (Delarive et al., 2003) and corneal implantation (Liu et al., 2006). Finally, the use of collagen as a scaffold for the development of a DDS has attracted the attention of many researchers all over the world (Wallace and Rosenblatt, 2003) for several applications, such as bone regeneration, eye, cardiac, and brain medicine (Lucas et al., 1989; Kaufman et al., 1994; Chiu et al., 2010; Chan et al., 2017) since the 1970s (Bradley and Wilkes, 1977).

### Functionalization of Collagen for Tissue Engineering Applications

**Figure 3:**
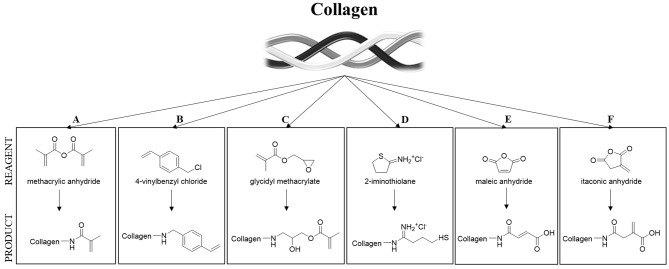
Strategies for collagen functionalization. **(A)** Methacrylic anhydride (Gaudet and Shreiber, 2012; Pupkaite et al., 2017); **(B)** 4-vinylbenzylchloride and **(C)** glycidyl methacrylate (Tronci et al., 2013); **(D)** thiol-functionalization (Holmes et al., 2017) and **(E,F)** unsaturated cyclic anhydrides (Potorac et al., 2014).

One of the most important limitations in using collagen-based materials in regenerative medicine applications remains their mechanical properties, which are often limited, especially at the viscoelastic level, specifically, for vTE, mechanical properties related to the high pressures and stresses encountered in the blood vessel (Achilli et al., 2010; Meghezi et al., 2015). Research has therefore focused on various ways of enhancing and controlling the polymerization, the stability in solutions, reducing enzymatic sensitivity, and controlling the pore size, in an attempt to increase mechanical strength. An interesting approach to maintain the structural integrity of a scaffold is to chemically, physically, or enzymatically cross-link the biopolymer (Davidenko et al., 2015; Liu et al., 2019). However, collagen has a limited number of functional groups (i.e., amine and carboxylic acids) that can enable cross-linking (Gallop and Paz, 1975; Rýglová et al., 2017). For this reason, cross-linkable modifications have been introduced on the protein structure (Ravichandran et al., 2016) (Figure 3). An overview of various types of modified collagen is shown in Table 2.

**Table 2:** Overview on various functional groups that have been introduced on the collagen backbone.

**Material**	**Functional group**	**Aim of the modification**	**Figure**	**References**
Collagen Type I	Methacrylate	Sutureless wound closure	A	Pupkaite et al., 2017
Collagen Type I	Methacrylate	Mechanically heterogeneous environments	A	Gaudet and Shreiber, 2012
Collagen Type I	4-vinylbenzyl chloride (4VBC) and glycidyl methacrylate (GMA)	Programmable macroscopic properties	B, C	Tronci et al., 2013
Collagen Type I	8-arm poly (ethylene glycol) norbornene-terminated (PEG-NB)	Injectable regenerative hydrogels	D	Holmes et al., 2017
Collagen Type I + III	Cyclic anhydrides	Mechanical performance enhancement	E, F	Potorac et al., 2014

## Collagen in vTE

### Collagen Coatings for Vascular Substitutes

One of the main complications related to the use of synthetic vascular grafts, and especially with the ones made of PET (Dacron), is linked to their high porosity and low elasticity. While porosity allows tissue ingrowth, ensuring a physiological integration of the implanted grafts, and a faster healing, it also causes excessive bleeding, inducing potential serious complications for the patients. Thus, the walls of the grafts must be rendered impermeable in order to avoid this outcome. For this reason, pre-clotting is a mandatory clinical step prior to the implantation of a Dacron (PET knitted or woven) graft. This technique consists in the conversion of the porous wall of the prosthesis into one that has been rendered impervious by reaction with blood (Yates et al., 1978). Despite helping in limiting bleeding, this technique is hampered by several disadvantages, such as the increase of the roughness of the luminal surface of the implanted grafts. This rougher surface increases the occurrence of turbulent blood flow and thrombus formation, and the increase in the rigidity of the graft straightforwardly diminishes their pliability.

The impregnation of porous Dacron vascular grafts with collagen was first proposed in the early 1960s (Humphries et al., 1961) as an alternative to pre-clotting. Striking improvements were obtained years later by Scott and colleagues in 1987 (Scott et al., 1987). Their bovine collagen-coated grafts did not require pre-clotting or special preparation and did not bleed once implanted in a canine model. The luminal surface of the grafts showed neointima formation, and the collagen coating was completely resorbed and substituted by native tissue after 3 months of implantation. Moreover, the collagen was non-thrombogenic or antigenic. That opened the door for the use of collagen-impregnated vascular grafts in the surgical treatment of aneurysms and for arterial bypass (Reigel et al., 1988; Freischlag and Moore, 1990; Noishiki et al., 1996), proving to be a viable alternative to the previously used pre-clotting technique, being able to compete equally against other proposed techniques and materials (Prager et al., 2003).

Nonetheless, these collagen-coated grafts have been demonstrated over the years not to be free from complications: Variable inflammatory response and tissue adhesion (Jonas et al., 1987), need of sustained chest drainage (Suehiro et al., 2003), and initiation of the immune response (Kobayashi et al., 1993) in the treated patients. Moreover, they showed no added value for the replacement of small-caliber arteries (Guidoin et al., 1996). However, the performances of the collagen-coated vascular grafts have stood the test of time, resulting in being one of the most used vascular grafts for medium- and large-diameter arteries substitution nowadays.

### Collagen-Based DDS

Biological signaling represents an important point in cell-driven tissue regeneration and providing signaling molecules greatly improves this process. However, when administering molecules and drugs, it is of crucial importance to reach the appropriate dose at a specific site and for the necessary period of time, in order to accomplish the desired effects. Thus, there is a need to release these molecules in a controlled way.

The development of collagen-based DDS for the release of pro-angiogenetic factors for wound healing applications and pro-endothelialization factors for vascular implant functionalization is highly sought after. Collagen has been widely studied as a biomaterial for DDS (Friess, 1998) and has found several uses in a variety of applications (Table 3).

**Table 3:** Collagen-based drug delivery systems.

	**Scaffold structure**	**Medical application**	**Biomolecule used**	**Cells seeded**	**References**
Growth factors/Drugs	Collagen sponges	Wound healing	VEGF	/	Schroeder et al., 2007
	Collagen sponges	Tissue regeneration	bFGF, HGF, PDGF-BB, VEGF, IGF-1, HB-EGF	/	Kanematsu et al., 2004
		Antibacterial	Gentamicin	/	Ivester et al., 2006
Genes	Collagen gels	Skin wound repair	PDGF A and B (genes)	/	Chandler et al., 2000
Cells	Electrospun collagen	Bone	/	BM-MSC	Shih et al., 2006
	Collagen–glycosaminoglycans scaffold	Cardiovascular	/	BM-MSC	Xiang et al., 2006
	Collagen sponges	Brain	/	NSC	Yu et al., 2010
	Collagen sponges and hydrogels	Intervertebral discs	/	Human intervertebral disc cells	Gruber et al., 2004, 2006

*bFGF, basic fibroblast growth factor; VEGF, vascular endothelial growth factor; HGF, hepatocyte growth factor; PDGF-BB, platelet-derived growth factor-BB; IGF-1, insulin-like growth factor-1; HB-EGF, heparin binding epidermal growth factor-like growth factor; BM-MSC, bone marrow mesenchymal stem cells; NSC, neural stem cells*.

The use of collagen-based DDS for vascular applications has been explored in recent years. Most of the studies performed aimed to increase the affinity for the collagen scaffolds toward ECs. The enrichment of collagen matrices with several pro-angiogenetic growth factors, such as vascular endothelial growth factor (VEGF) (Steffens et al., 2004; Koch et al., 2006; He et al., 2011), stromal derived factor-1 alpha (SDF-1α) (Laiva et al., 2018), and basic fibroblast growth factor (bFGF) (Hao et al., 2018), has shown promising results in terms of controlling the release of the loaded molecules and the angiogenesis induction, which in turn results in compelling effects during wound repair and for tissue engineering applications.

As mentioned in the Introduction, the use of synthetic vascular grafts for the treatment of occlusive vascular diseases is still a burden by grafts failure, mainly caused by thrombosis and neointima hyperplasia. Implants modifications using pro-endothelialization molecules and growth factors with the aim of speeding up the re-endothelialization process have been proposed over the last years to guide the optimal integration of the grafts and to overcome the aforementioned problems. The use of vascular graft enrichment has also been investigated. In their work from 2000, Wissink et al. developed a heparinized, cross-linked collagen matrix for the controlled release of bFGF to improve the endothelialization of vascular grafts (Wissink et al., 2000). They were able to improve the binding of the loaded bFGF to the heparinized cross-linked matrix and to release it in a controlled way over time, leading to an improvement in the proliferation of treated EC *in vitro*.

The occurrence of infections in newly implanted synthetic vascular grafts is one of the complications that may arise, hampering the functionality of the prosthesis. Conventional treatments of vascular graft infections consist in the excision of the infected graft with extra anatomic bypass grafting (Yeager et al., 1999). To avoid the need of another surgical operation to treat the infected grafts, the use of DDS has been proposed. In particular, collagen-based matrices have been demonstrated to be effective in delivering antibiotic agents to limit and treat bacterial infections in implanted synthetic vascular grafts (Chervu et al., 1991; Batt et al., 2003; Schneider et al., 2008; Herten et al., 2017), avoiding the need for subsequent surgical interventions.

### Collagen Scaffolds for Vascularization and Artificial Blood Vessel Development

Over the years, collagen has been used as a pro-vascularization scaffold for several applications. In fact, the ability of collagen scaffolds to support angiogenesis and the formation of neo-vasculature has been demonstrated (Nicosia et al., 1991). Collagen scaffolds have been first used as an *in vitro* model for the study of the angiogenetic process (Vernon et al., 1995), but their use has been shortly translated to the clinic (Abraham et al., 2000) for several applications. In 2008, Shen et al. showed how a VEGF-modified collagen scaffold was able to efficiently promote penetration, proliferation, and assembly of ECs in the scaffold (Shen et al., 2008). In 2016, Chan and colleagues developed a 3D scaffold from bovine collagen type I able to support capillary formation *in vivo* and vascularization once implanted in animal models (Chan et al., 2016). Similarly, other groups demonstrated how implanted collagen scaffolds were able to promote EC infiltration and vascularization (Cherubino et al., 2016; Wahl et al., 2016). Interestingly, the joint use of other ECM components along collagen, like elastin or glycosaminoglycans, has been shown to exert different effects on the vascularization of collagen scaffolds (Schmidt et al., 2017).

Collagen is one of the most abundant proteins in the vascular ECM. There, collagen fibers limit the distension of the vessel and provide attachment for SMCs, allowing them to transmit circumferential forces to the vessel wall, ultimately conferring excellent mechanical support to the blood vessel wall (Bou-Gharios et al., 2004). Therefore, the use of collagen, in particular type I, as a scaffold in the development of tissue-engineered vascular substitutes has been largely explored. The first use of collagen gels to manufacture a vascular substitute dates back to 1986, when Weinberg and Bell attempted to reconstitute a blood vessel (Weinberg and Bell, 1986). Their method consisted in the production of a multilayered tubular construct made of collagen seeded with SMCs and fibroblasts and of the endothelialization of its lumen. Despite showing very low mechanical properties and the impossibility to be used for clinical purposes, this work marked a major advance in the field of vTE, with several groups following in the footsteps (Hirai et al., 1994; Seliktar et al., 2000; Boccafoschi et al., 2007) and trying to improve the system. One of the main problems related to this kind of construct is its mechanical properties. Different variants of the methodology from Weinberg and Bell, such as winding leaflets around a mandrel to promote compaction of collagen (L'Heureux et al., 1993), magnetic pre-alignment of collagen fibers to increase tensile strength (Tranquillo et al., 1996), cross-linking of collagen scaffolds by glycation (Girton et al., 2000), or ultraviolet radiation (Charulatha and Rajaram, 2003) have been developed to improve the mechanical properties of the substitutes. However, the extent of these improvements still does not allow the implantation and, thus, the use in the medical practice of these grafts. The seeded cells play an important role too: SMCs have been demonstrated to actively influence the compaction of the collagen scaffold (Berglund et al., 2003; Meghezi et al., 2015) and to align along the direction of the collagen fibers (Hirai et al., 1994), helping in increasing the mechanical properties of the substitutes. The biological properties have also been studied. Different molecules have been used to modulate the cellular response toward these scaffolds. The addition of insulin and growth factors, such as TGF-β makes it possible to increase collagen production by the seeded cells (Long and Tranquillo, 2003), and the addition of dermatan sulfate has been able to increase the endothelialization of the lumen and, as a result, to reduce platelet adhesion and activation (Matsuda et al., 1988). In recent years, hybrid collagen vascular substitutes containing both synthetic (He et al., 2005; Stitzel et al., 2006; Jeong et al., 2007) and natural polymers, such as fibrin (Cummings et al., 2004) and elastin in particular (Berglund et al., 2004; Koens et al., 2010, 2015), have been developed to further increase the mechanical and biological properties of the collagen-based vascular grafts, aiming to obtain an artificial vessel as close as possible to the natural ones.

### Pre-clinical and Clinical Studies of Collagen for vTE Applications

As of today, the main use of collagen for clinical applications is as replacement scaffolds (i.e., tissue fillers) and as support matrices (i.e., matrix rich tissues). Collagen scaffolds used in clinical practice primarily include skin substitutes and dermal fillers. However, the use of collagen for other applications, including vascular applications, is increasing. In fact, a number of positive factors indicate that the use of a collagen-based product is becoming an attracting prospective for vTE purposes (Dogan et al., 2017). Table 4 shows some of the pre-clinical studies conducted on collagen-based vTE products. It can be observed that collagen-based materials for vascular applications, especially for vascular grafts, are successfully used in pre-clinical studies involving *in vivo* testing and, therefore, physiological stimulation. It can be concluded that research in the field is moving toward the achievement of those optimal properties needed for the clinical translation.

**Table 4:** Pre-clinical and clinical studies on collagen-based vascular tissue engineering products.

**Material**	**Structure**	**Application**	**Implanted in**	**References**
Bovine collagen type I	Porous collagen scaffolds	Tissue vascularization	Murine model (C57B/L6 mice)	Chan et al., 2016
Rat tail type I collagen	Dense gel tubes	Small-diameter vascular grafts	Murine model (Sprague–Dawley rats)	Li et al., 2017
Autologous collagen matrix	*in vivo* tissue-engineered autologous vascular graft	Pediatric pulmonary artery augmentation	Human model (2-years-old girl with pulmonary atresia)	Kato et al., 2016
Collagen type I and type III	Porous collagen membranes	Myocardial ischemia repair	Rabbit model	Gao et al., 2011

## Collagen-Based Pseudo-Physiological Models for Cardiovascular Therapy Development

### Development of 3D *in vitro* Models for Cardiovascular Research

Although tissue-engineered blood vessels as living arterial substitutes have been studied extensively in the last 25 years, clinical translation has not yet happened (Zhang et al., 2007; Nemeno-Guanzoni et al., 2012). The mechanism by which these grafts integrate into the host's circulatory system and remodel into functional blood vessels remains unclear (Pashneh-Tala et al., 2016). Despite this drawback, the vTE grafts can be used as an advanced model of the vascular wall for the *in vitro* testing of drugs and devices. In fact, currently used *in vitro* pre-clinical models represent an overly simplified vascular environment, not able to reproduce the complex cell–cell and cell–environment interactions taking place *in vivo*. On the other hand, *in vivo* animal models currently used for the development of medical drugs and devices show limitations and disadvantages, such as animal-to-human variations in anatomy, physiology, and functions together with high costs and ethical burden (Byrom et al., 2010; Swartz and Andreadis, 2013). The four main factors to consider in order to develop a successful *in vitro* vascular wall model are as follows: (i) a scaffold that can support cell growth, (ii) an appropriate cell population, (iii) the right biological (use of biomolecules, such as growth factors), and (iv) mechanical stimuli to influence the proper development of the construct (Fortunato et al., 2017). Different research groups have been working with the final aim to develop *in vitro* models able to finely mimic the wall structure of a healthy human artery. Some examples of the development in *in vitro* models, based on different approaches, can be found in Table 5.

**Table 5:** Vascular tissue-engineered *in vitro* models and strategies used.

**Developed model**	**Strategy**	**References**
Planar vessel wall model	Collagen type I hydrogel	Loy et al., 2016
Tissue-engineered vascular equivalent	Polyglycolic-acid (PGA) meshes	Robert et al., 2013
Tubular vascular model for inflammatory response analysis	Collagen type I Scaffold	Chen et al., 2018
Micro-vascular networks	3-D printing approach	Schoneberg et al., 2018

Collagen is widely used for the development of physiologically relevant *in vitro* models (Boccafoschi et al., 2005; Seifu et al., 2013; Pawelec et al., 2016). One of the main challenges in developing an *in vitro* vascular wall model is the interaction between the different populations of cells (Battiston et al., 2014). Loy et al. (2016, 2018) developed an *in vitro* model of the vascular wall based on collagen gels cellularized with SMCs, fibroblasts, and ECs. In this study, the importance of co-culturing these three vascular cell types in order to promote cell–matrix remodeling and to obtain an early expression of elastic fiber-related proteins was stressed. Furthermore, it was shown that the use of a tri-culture model resulted in cell–cell interactions similar to *in vivo* conditions. Another challenge in the development of advanced *in vitro* vascular models, as for vTE grafts, is the improvement of mechanical properties (i.e., compliance, burst pressure, and elasticity) and an increase in complexity of the model. Pezzoli et al. (2018) developed a collagen-based *in vitro* model that was supplemented with human plasma fibronectin. This resulted in an increase in elastin deposition by SMCs, as well as an increase in the expression levels of several proteins required for elastogenesis (i.e., fibrillin-1, lysyl oxidase, fibulin-4, and latent TGF-β binding protein-4). The study showed how fibronectin plays a crucial role in the production of physiological-like, elastin-containing collagen matrices displaying superior mechanical properties compared to the currently used models. It has been shown that *in vitro* simulation of physiological biochemical and biomechanical conditions plays a crucial role in the development of a physiologically relevant model of the vascular wall. To achieve this, research has focused on different strategies, including the use of bioreactors (Bono et al., 2017; Tresoldi et al., 2017). Bioreactors have gained large interest because they provide the possibility to mimic a physiological environment similar to the human *in vivo* situation, allowing the improvement of both mechanical and biological properties of *in vitro* models (Arslan-Yildiz et al., 2016; Tresoldi et al., 2017; Loy et al., 2018). The physiological-like mechanical stimulation is of utmost importance in the development of an engineered model of the vascular wall. The applied hemodynamic forces can lead to improvements in the structural and mechanical properties of the engineered construct. This is mainly due to an increased circumferential orientation of the SMCs and the alignment of the ECs along the flow direction, leading to a higher yield stress, ultimate stress, and elastic modulus (Ziegler et al., 1995; Tresoldi et al., 2017). Moreover, the simulation of physiological pulsatile perfusion improves not only the artificial vascular development in terms of cell alignment and organization (Houtchens et al., 2008; Lesman et al., 2016; Asano et al., 2018), but also the cell differentiation and phenotypic maintenance (Cevallos et al., 2006; Li and Xu, 2007; Qiu et al., 2014), ECM production (Stanley et al., 2000; Halka et al., 2008), vascular tone (Garoffolo et al., 2018), and mechanical properties (Seliktar et al., 2000) of the engineered construct (Meghezi et al., 2012; Wissing et al., 2017;Colunga and Dalton, 2018).

### Currently Used Collagen-Based *in vitro* Models for CVDs and Drug Development Studies

Medical drugs that contribute to blood pressure elevation or reduction can have a great efficacy in reducing cardiovascular risks (Cameron et al., 2016). Vasodilation and vasoconstriction directly affect the blood vessel diameter and thus an increased or decreased blood flow; therefore, they have an immediate impact on the blood pressure (Toda et al., 2013). More than 80% of currently proposed pharmaceutical drug candidates that enter clinical trials fail due to concerns with human efficacy and toxicity (Fernandez et al., 2016). Animal responses to drugs exhibit differences in toxic doses and drug metabolism. Therefore, the development of *in vitro* models that accurately mimic specific biological interactions, particularly relevant to diseases, using human cells to be able to predict local responses to administered drugs is of critical importance (Truskey and Fernandez, 2015; Fernandez et al., 2016; Ronaldson-Bouchard and Vunjak-Novakovic, 2018). For example, it is known that the SMCs in the media layer of the vascular wall are fundamental for the regulation of the vascular tone, being a key factor in the contractile portion of the vascular wall (Wolf et al., 2016). Next to this, the ECs layer exerts important effects on the vascular tone too, mainly through the release of vasoconstrictor and vasodilator molecules (Toda et al., 2013). Vaso-activity, being the vascular activity involving the effect of either increasing or decreasing blood pressure and/or heart rate, is considered an important feature and a desirable characteristic for a tissue-engineered model. It is influenced by many factors including cell phenotype and cell–matrix interactions. Different models have been developed over the years. The group of Laflamme (Laflamme et al., 2005) made use of a simple SMC-based media layer for studying the vaso-reactive properties, whereas Fernandez et al. (2016) and Niklason et al. (1999) fabricated a model based on a media layer combined with an EC layer to mimic the vessel intima layer.

Fernandez et al. (2016) validated the use of non-destructive monitoring strategies on collagen-based vascular constructs. This strategy helped in discovering that acetylcholine, which stimulates the release of nitric oxide, prostacyclin, and endothelium-derived hyperpolarization factor in vessels with a healthy and intact endothelium, is an important vasodilator in coronary arteries, enabling the quantification of endothelium-dependent vasodilation. On the other hand, phenylephrine enables the non-destructive measure of endothelium-independent vasoconstriction. The group of Schutte has studied the functionality of collagen-based engineered vascular media layers by looking at a large panel of vasoactive agents that consists of drugs from both intrinsic and extrinsic pathways (Schutte et al., 2010b). The study has shown that the collagen-based models were capable of generating a measurable response to several different vasoconstrictors and vasodilators. They highlighted the importance of vaso-activity and the functionality of developed models, as well as the choice of a large panel of drugs to test both features. In their work from 2016, Wolf et al. gave an overview on different engineered vascular constructs studied for pharmacological studies (Wolf et al., 2016). These studies demonstrate that TE vascular constructs can be used as *in vitro* models to investigate pharmacologically induced responses. However, these studies have currently been done on simplified models of the vascular wall using only the media and intima layer. Further research on the evaluation of the effects of vaso-reactive stimuli on a more advanced, complex, and physiologically relevant model of the vascular wall is yet to be studied. It can be concluded that *in vitro* models of the vascular wall show great potential and importance in the study of CVD and treatment, both at pre-clinical and clinical stages.

## Discussion

### Strengths, Weaknesses, Opportunities, and Threats of Collagen as a Biomaterial for vTE

**Figure 4:**
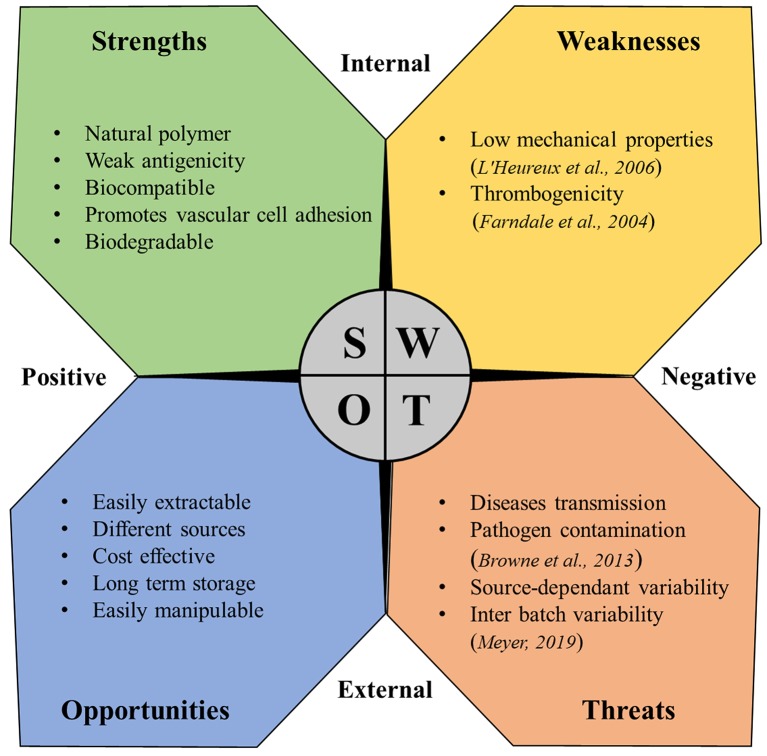
SWOT analysis for collagen as a material for vascular tissue engineering (Farndale et al., 2004; L'Heureux et al., 2006; Browne et al., 2013; Meyer, 2019).

Despite the multiple beneficial properties and the variety of proposed applications in vTE described in this review, the use of collagen in vascular medicine is still hampered by some problems. The strength, weaknesses, opportunities, and threats (SWOT) analysis represented in Figure 4 summarizes the benefits and the main problems and concerns related to the use of collagen in this field.

### Limits of Collagen

As mentioned in the SWOT analysis, two main limits heavily hamper the use of collagen in vTE: collagen thrombogenicity and its poor mechanical properties. Especially for applications where blood contact plays a major role like in vTE, collagen intrinsic thrombogenicity represents a major limitation. In fact, collagen is known to be one of the major activators of platelet response, being able to trigger and support both platelet adhesion and activation (Farndale et al., 2004), thus impacting the thrombogenicity of vascular devices. Thrombogenic potential, especially for vascular graft, is a major issue, being responsible for earlier graft occlusion (Sarkar et al., 2007). Thus, the use of collagen has been addressed for these reasons (Guidoin et al., 1996). Modification of the collagen through bonding of antithrombotic agents, such as heparin has been proposed over the years (Keuren et al., 2004; Scharn et al., 2006; Al Meslmani et al., 2014), partially solving the issue but leaving an open problem. Collagen plays a major role for *in vivo* vascular stiffness, conferring mechanical resistance along with the other molecules of the vascular ECM. However, extraction processes critically compromised the mechanical strength of collagen. As a consequence, low mechanical properties are reported as one of the main problems related to collagen for vTE (L'Heureux et al., 2006), thus limiting its clinical application. Over the years, improvements have been shown through dynamic conditioning (Seliktar et al., 2000; Buttafoco et al., 2006; Schutte et al., 2010a) or enhanced cross-linking techniques (Brinkman et al., 2003). Unfortunately, although these are promising techniques, all reported cases in the literature show ultimate mechanical properties significantly below those of native blood vessels (Pashneh-Tala et al., 2016), once again showing the difficulties in the clinical translation.

## Conclusions and Outlook

Collagen-based scaffolds have been proven to be a versatile biomaterial for vascular applications, gaining great achievements in vTE. Although collagen is complex by nature, its use allowed great developments in implants and drug delivery and offers great opportunities in several fields of tissue engineering, for dermal, cardiovascular, and connective applications. From a scientific point of view, the open challenge remains to be able to reproduce the hierarchically complex nature of tissues starting from collagen. In fact, in living tissue, a number of biologically active molecules, proteins, and cells work together in a very dynamic environment continuously orchestrating regeneration. From an industrial point of view, although some companies are now able to extract, sterilize, de-immunize, neutralize, and finally provide different types of collagen in a reproducible manner, its cost remains prohibitive and seriously limits studies and developments in the field. Therefore, the open challenges remain to find alternative sources and to optimize processes and protocols for reliable, reproducible, safe, and low-cost collagen. Finally, accreditation and regulatory bodies are the missed elements in this complex equation. The idea to synthesize collagen in laboratory is an idea worthy to be further explored and that will also facilitate the regulation concerning the collagen structures, in the interest of the patients, and for the benefit of the society.

## Author Contributions

FC and DM conceived the layout, the rationale, and the plan of this manuscript. FC and NP wrote the first draft of the manuscript that was iteratively improved by SV, FB, and DM.

### Conflict of Interest Statement

The authors declare that the research was conducted in the absence of any commercial or financial relationships that could be construed as a potential conflict of interest.

## Footnotes

^1^World Health Organization. *Top 10 Causes of Death*. Available online at: http://www.who.int/gho/mortality_burden_disease/causes_death/top_10/en/ (accessed January 28, 2019).
